# Epidemiological information is key when interpreting whole genome sequence data – lessons learned from a large *Legionella pneumophila* outbreak in Warstein, Germany, 2013

**DOI:** 10.2807/1560-7917.ES.2017.22.45.17-00137

**Published:** 2017-11-09

**Authors:** Markus Petzold, Karola Prior, Jacob Moran-Gilad, Dag Harmsen, Christian Lück

**Affiliations:** 1 Institute of Medical Microbiology and Hygiene, Dresden University of Technology, Dresden, Germany; 2 These authors contributed equally to the work; 3 The ESCMID Study Group for Legionella infections (ESGLI); 4 Department for Periodontology and Restorative Dentistry, University Hospital Muenster, Muenster, Germany; 5 Public Health Services, Ministry of Health, Jerusalem, Israel; 6 Faculty of Health Sciences, Ben-Gurion University of the Negev, Beer-Sheva, Israel

**Keywords:** Legionella pneumophila, multilocus sequence typing, Legionnaires' disease, Whole Genome Sequencing, cgMLST, Outbreaks

## Abstract

Whole genome sequencing (WGS) is increasingly used in Legionnaires’ disease (LD) outbreak investigations, owing to its higher resolution than sequence-based typing, the gold standard typing method for *Legionella pneumophila,* in the analysis of endemic strains. Recently, a gene-by-gene typing approach based on 1,521 core genes called core genome multilocus sequence typing (cgMLST) was described that enables a robust and standardised typing of *L. pneumophila*. **Methods**: We applied this cgMLST scheme to isolates obtained during the largest outbreak of LD reported so far in Germany. In this outbreak, the epidemic clone ST345 had been isolated from patients and four different environmental sources. In total 42 clinical and environmental isolates were retrospectively typed. **Results**: Epidemiologically unrelated ST345 isolates were clearly distinguishable from the epidemic clone. Remarkably, epidemic isolates split up into two distinct clusters, ST345-A and ST345-B, each respectively containing a mix of clinical and epidemiologically-related environmental samples. **Discussion/conclusion**: The outbreak was therefore likely caused by both variants of the single sequence type, which pre-existed in the environmental reservoirs. The two clusters differed by 40 alleles located in two neighbouring genomic regions of ca 42 and 26 kb. Additional analysis supported horizontal gene transfer of the two regions as responsible for the difference between the variants. Both regions comprise virulence genes and have previously been reported to be involved in recombination events. This corroborates the notion that genomic outbreak investigations should always take epidemiological information into consideration when making inferences. Overall, cgMLST proved helpful in disentangling the complex genomic epidemiology of the outbreak.

## Introduction

*Legionella* spp. is the causative agent of Legionnaires’ disease (LD) named after its first occurrence during a convention of the American Legion in 1976 [1,2]. These rod shaped Gram-negative bacteria inhabit all kinds of natural and man-made fresh water reservoirs including cooling towers (CT), spas and drinking water systems. Inhalation of legionellae-containing aerosols originating from contaminated environmental reservoirs is the main route of infection. However, a case of a person-to-person transmission of *Legionella* under special circumstances was recently reported [3].

LD accounts for 2–20% of community-acquired pneumonia (CAP) and the number of cases in Europe reached almost 6,000 in 2014 [4]. In Germany ca 1,000 cases are reported annually, representing an incidence of ca 11 cases per million population [5]. In Europe ca 10 % of the cases are related to clusters or outbreaks. So far, 60 species and more than 70 serogroups (Sg) of the genus *Legionella* were reported from which around half were implicated in human infections [6]. The vast majority of LD cases is caused by *L. pneumophila* serogroup Sg1 isolates, especially monoclonal antibody (mAb) 2/3–1 positive strains [7]. Hitherto, all CT-related outbreaks reported worldwide were caused by these subtypes [8].

Molecular and serological typing methods are predominately applied to the species *L. pneumophila*. The two well-established epidemiological typing methods for comparison of clinical and environmental isolates consist of the subgrouping scheme based on mAbs and the sequence based typing (SBT) method, an adapted multilocus sequence typing (MLST) variant that defines sequence types (ST) [9,10]. Other methods have been described but lack uniform interpretation of results in inter-laboratory comparison studies [11,12].

Currently, SBT is the gold standard to genotype *L. pneumophila* isolates. The allelic profile of seven genes enables the assignment of an ST to the corresponding isolate. A database, curated by Public Health England (PHE), London, United Kingdom, in cooperation with the European Centre for Disease Prevention and Control (ECDC), Stockholm, Sweden, facilitates the exchange of typing data and can be queried for surveillance and epidemiological studies of *L. pneumophila* [10]. Currently, the database consists of more than 11,000 reported isolates with 2,298 different STs (status as of 05 January 2017). Despite the index of discrimination of the SBT scheme being around 0.92, typing of frequently circulating STs with this method, e.g. ST1, ST47 and ST23, proves less informative to further differentiate strains within these rather big clonal groups [13]. An additional typing step is thus needed, but modalities attempted this far such as spoligotyping were of limited value [14].

Due to a higher level of discrimination compared with gold standard typing methods of different bacteria, including *L. pneumophila,* whole genome sequencing (WGS) has become a frequently applied tool in outbreak investigations [15-20]. While use of this tool has mostly relied on analysis of single nt polymorphisms (SNPs), a few studies are based on a genome-wide gene-by-gene allele calling approach for *L. pneumophila* Sg1 strains. These extended MLST schemes enable a detailed comparison of two or more isolates by either considering all genes of a species (pangenome) in what is called whole genome MLST (wgMLST), or alternatively, a set of conserved genes of a species, namely core genome MLST (cgMLST) [21]. Analysis of several related strains and strains that were involved in small outbreaks using these cgMLST or wgMLST produced results that were in agreement with current standard typing methods, indicating the suitability of these methods as typing tools for *L. pneumophila* Sg1 isolates [18,19,22].

Here, we report in detail the retrospective application of a previously described cgMLST scheme consisting of 1,521 genes [18], to the largest outbreak of LD reported so far in Germany, in order to validate this scheme on a large and homogenous set of isolates. The outbreak occurred in the summer of 2013 in the city of Warstein. In total, 78 confirmed LD cases were reported and multiple potential environmental sources of infection carrying the outbreak strain were implicated. These included several CTs, municipal and private waste water treatment plants (WWTPs) and the river Wester, which runs through the city of Warstein. The outbreak strain was characterised as *L. pneumophila* Sg1, mAb-subgroup Knoxville, ST345 [23].

## Methods

### Cultivation of *Legionella pneumophila* isolates and DNA extraction

Respiratory samples (bronchoalveolar lavages, BAL) from outbreak patients, with and without heat treatment at 50 °C for 30 min were plated on non-selective buffered charcoal-yeast extract (BCYE) agar and a selective agar containing cefamandole, polymyxin B, and anisomycin (BMPA) and incubated at 36 °C in humidified atmosphere supplemented with 5% CO_2_. Isolated strains were initially serotyped by using a latex agglutination test (Oxoid, Wesel, Germany) and confirmed by using the Dresden panel of mAbs as described elsewhere [9]. The environmental isolates were isolated according to ISO11731/1998 [24,25] and typed in a similar way. Additionally, all samples were typed according the *L. pneumophila* SBT protocol [10].

DNA from respiratory samples was extracted using the EZ1 DNA tissue kit (Qiagen, Hilden, Germany) according to manufacturer instructions. Clinical samples were tested with a *L. pneumophila* specific PCR (DUPLICα RealTime Legionella pneumophila Kit, Euroclone, Milan, Italy) and a *L. pneumophila* Sg1 specific PCR [26]. Furthermore, direct genotyping from three culture negative PCR-positive clinical samples was attempted using the nested SBT (nSBT) protocol [27].

### Whole genome sequencing and assembly

Deep frozen clinical and environmental isolates collected during the outbreak (stored in 15% glycerol at − 80 °C) were thawed, sub-cultured on BCYE-agar plates (Oxoid, Wesel, Germany), and incubated for another 48 hour as described above. We additionally included unrelated isolates of ST345 as well as two strains of *L. pneumophila* Sg1 mAb-subgroup Knoxville ST600, a double locus variant of ST345 frequently isolated during the outbreak (Table 1). Colonies were harvested and resuspended in sterile distilled water for subsequent DNA extraction using the purification protocol for Gram-negative bacteria of the MagAttract HMW DNA Kit (Qiagen, Hilden, Germany).

**Table 1:** *Legionella pneumophila* serogroup 1 samples and isolates used for the retrospective analysis of a 2013 Legionnaires’ disease outbreak by core genome multilocus sequence typing, Germany (n = 45)

Sample ID / source (C/E)^a^	Epidemiological context to outbreak	Culture	Monoclonal subgroup^b^	ST^c^	Allelic profile (*flaA*, *pilE*, *asd*, *mip*, *mompS*, *proA*, *neuA*)	Outbreak cluster^d^
L13–435 (C)	Warstein outbreak	+	Knoxville	345	6, 10, 19, 3, 19, 4, 11	ST345-B
L13–438 (C)	Warstein outbreak	+	Knoxville	345	6, 10, 19, 3, 19, 4, 11	ST345-B
L13–439 (C)	Warstein outbreak	+	Knoxville	345	6, 10, 19, 3, 19, 4, 11	ST345-B
L13–444 (C)	Warstein outbreak	+	Knoxville	345	6, 10, 19, 3, 19, 4, 11	ST345-A
L13–445/-446 (C)^e^	Warstein outbreak	+	Knoxville	345	6, 10, 19, 3, 19, 4, 11	ST345-A
L13–473 (C)	Warstein outbreak	+	Knoxville	345	6, 10, 19, 3, 19, 4, 11	ST345-B
L13–477 (C)	Warstein outbreak	+	Knoxville	345	6, 10, 19, 3, 19, 4, 11	ST345-A
W13–845–1 (E)	Cooling tower, source A, Warstein outbreak	+	Knoxville	600	6, 10, 19, 28, 19, 4, 11	NA^f^
W13–845–4 (E)	Cooling tower, source A, Warstein outbreak	+	Knoxville	345	6, 10, 19, 3, 19, 4, 11	ST345-A
W13–845–8 (E)	Cooling tower, source A, Warstein outbreak	+	Knoxville	600	6, 10, 19, 28, 19, 4, 11	NA^f^
W13–871–1 (E)	Condenser, source B, Warstein outbreak	+	Knoxville	345	6, 10, 19, 3, 19, 4, 11	ST345-A
W13–873–1 (E)	Pump shaft, source A, Warstein outbreak	+	Knoxville	345	6, 10, 19, 3, 19, 4, 11	ST345-A
W13–874–15 (E)	Pump shaft, source A, Warstein outbreak	+	Knoxville	345	6, 10, 19, 3, 19, 4, 11	ST345-A
W13–875–15 (E)	River inlet, source A, Warstein outbreak	+	Knoxville	345	6, 10, 19, 3, 19, 4, 11	ST345-A
W13–875–17 (E)	River inlet, source A, Warstein outbreak	+	Knoxville	345	6, 10, 19, 3, 19, 4, 11	ST345-A
W13–876–13 (E)	Aeration basin, source C, Warstein outbreak	+	Knoxville	345	6, 10, 19, 3, 19, 4, 11	ST345-A
W13–878–1 (E)	River water, source D, Warstein outbreak	+	Knoxville	345	6, 10, 19, 3, 19, 4, 11	ST345-A
W13–879–1 (E)	River water, source D, Warstein outbreak	+	Knoxville	345	6, 10, 19, 3, 19, 4, 11	ST345-A
W13–952–4 (E)	Pre-sedimentation basin, source B, Warstein outbreak	+	Knoxville	345	6, 10, 19, 3, 19, 4, 11	ST345-A
W13–953–3 (E)	Pre-sedimentation basin, source B, Warstein outbreak	+	Knoxville	345	6, 10, 19, 3, 19, 4, 11	ST345-B
W13–953–4 (E)	Pre-sedimentation basin, source B, Warstein outbreak	+	Knoxville	345	6, 10, 19, 3, 19, 4, 11	ST345-B
W13–954–3 (E)	Pre-sedimentation basin, source B, Warstein outbreak	+	Knoxville	345	6, 10, 19, 3, 19, 4, 11	ST345-B
W13–957–2 (E)	Outlet, source B, Warstein outbreak	+	Knoxville	345	6, 10, 19, 3, 19, 4, 11	ST345-A
W13–959–3 (E)	Aeration basin, source C, Warstein outbreak	+	Knoxville	345	6, 10, 19, 3, 19, 4, 11	ST345-B
W13–959–4 (E)	Inlet from source B, source C, Warstein outbreak	+	Knoxville	345	6, 10, 19, 3, 19, 4, 11	ST345-B
W13–1093 (E)	Cooling tower, source A, Warstein outbreak	+	Knoxville	345	6, 10, 19, 3, 19, 4, 11	ST345-A
W13–1096–2 (E)	Cooling tower, source A, Warstein outbreak	+	Knoxville	345	6, 10, 19, 3, 19, 4, 11	ST345-A
W14–178 (E)	Pre-sedimentation basin, source B, Warstein outbreak	+	Knoxville	345	6, 10, 19, 3, 19, 4, 11	ST345-A
W14–472 (E)	Pre-sedimentation basin, source B, Warstein outbreak	+	Knoxville	345	6, 10, 19, 3, 19, 4, 11	ST345-A
W14–474 (E)	Pre-sedimentation basin, source B, Warstein outbreak	+	Knoxville	345	6, 10, 19, 3, 19, 4, 11	ST345-A
W14–476 (E)	Pre-sedimentation basin, source B, Warstein outbreak	+	Knoxville	345	6, 10, 19, 3, 19, 4, 11	ST345-B
W14–489 (E)	Pre-sedimentation basin, source B, Warstein outbreak	+	Knoxville	345	6, 10, 19, 3, 19, 4, 11	ST345-A
P13–308 (C)	Clinical sample, Warstein outbreak	-	ND^f^	345	6, 10, 19, 3, 19, 4, 11	NA^g^
P13–402 (C)	Clinical sample, Warstein outbreak	-	ND^f^	ND	6, 10, 0, 3, 0, 4, 11	NA^g^
P13–733 (C)	Clinical sample, Warstein outbreak	-	ND^f^	345	6, 10, 19, 3, 19, 4, 11	NA^g^
EULV1461 (C)	Unrelated isolate (France)	+	Knoxville	345	6, 10, 19, 3, 19, 4, 11	NA^g^
EULV1647 (E)	Unrelated isolate (the Netherlands)	+	Knoxville	345	6, 10, 19, 3, 19, 4, 11	NA^g^
EULV1654 (C)	Unrelated isolate (the Netherlands)	+	Knoxville	345	6, 10, 19, 3, 19, 4, 11	NA^g^
EULV3674 (C)	Unrelated isolate (the Netherlands)	+	Knoxville	345	6, 10, 19, 3, 19, 4, 11	NA^g^
EULV5358 (C)	Unrelated isolate (France)	+	OLDA	345	6, 10, 19, 3, 19, 4, 11	NA^g^
EULV6345 (C)	Unrelated isolate (France)	+	Knoxville	345	6, 10, 19, 3, 19, 4, 11	NA^g^
EULV9125 (C)	Unrelated isolate (France)	+	Knoxville	345	6, 10, 19, 3, 19, 4, 11	NA^g^
Corby (C)	Unrelated isolate (United Kingdom)	+	Knoxville	51	6, 10, 15, 28, 9, 14, 6	NA^g^
Alcoy 2300/99 (C)	Unrelated isolate (Spain)	+	Knoxville	578	6, 10, 15, 13, 9, 14, 6	NA^g^

C: clinical sample; E: environmental sample; ID: identity; NA: not applicable; ND: not determined; ST: sequence type.

^a^ Source of sample: C corresponds to clinical isolates; E corresponds to environmental isolates.

^b^ Monoclonal subgrouping as described by Helbig et al. [9].

^c^ Sequence-based typing by using Sanger sequencing according to the ESCMID Study Group for *Legionella* Infections (ESGLI) protocol [10].

^d^ Outbreak ST345 strain cluster assignment by core genome multilocus sequence typing.

^e^ Isolates L13–445/-446 were isolated from the same patient.

^f^ The molecular antibody-subgroup and/or sequence type were/was not determined because no isolate was obtained and/or because of incomplete direct sequence-based typing.

^g^ Cluster assignment within the ST345 outbreak strain was not applicable when the isolate was from a chosen non-outbreak reference strain, when the isolate had another ST than ST345, or when no isolate could be obtained.

Sequencing libraries were prepared using the Nextera XT library prep kit (Illumina GmbH, Munich, Germany) for a 250 bp paired-end sequencing run on an Illumina MiSeq sequencer. Samples were sequenced to aim for a minimum 100-fold coverage using Illumina’s recommended standard protocols with dual-index barcoding and rotation of barcodes over time. Sequencing run quality (Q30 and output) had to fulfil the manufacturer’s minimum specifications. The resulting FASTQ files were quality trimmed and assembled de novo using the Velvet assembler that is integrated in Ridom SeqSphere ^+^  v.3.0 software (Ridom GmbH, Münster, Germany) [28]. Here, reads were trimmed at their 5'- and 3'-ends until an average base quality of 30 was reached in a window of 20 bases, and the assembly was performed with Velvet version 1.1.04 [29] using optimised k-mer size and coverage cut-off values based on the average length of contigs with > 1,000 bp.

### Core genome multilocus sequence typing (cgMLST) analysis

A cgMLST was performed using SeqSphere ^+^  with the *L. pneumophila* typing scheme described by Moran-Gilad et al. [18]. This scheme includes 1,521 core genome genes and the basic local alignment search tool (BLAST)-based allele calling procedure details have been described previously [18]. The percentage of good cgMLST targets determined the overall sequence quality of every sample such that samples containing at least 95% of extracted cgMLST targets were considered typeable. Alleles for each gene were assigned automatically by the SeqSphere ^+^  software to ensure a unique nomenclature. The combination of all alleles in each strain formed an allelic profile that was used to generate minimum spanning trees (MST). Targets with missing values in one of the strains compared were omitted during distance calculation. In order to maintain backwards compatibility with *L. pneumophila* SBT, sequences of the seven genes comprising the allelic profile of the SBT schemes were separately extracted from finished genomes and WGS data and then queried against the SBT database [10] in order to assign classic STs in silico.

### Detection of recombined regions

The de novo assembly FASTA contig files of four samples were chosen (L13–435, L13–473, W13–879–1, and W13–952–4) in order to analyse their genomes for putative recombined regions. Mauve (version 20150226 build 10, default parameters) [30] was used to calculate a multiple alignment of the four genomes. A SeqSphere ^+^  function was used to convert the Mauve alignment file from XMFA format into a FASTA and thereby concatenating the alignments for each of the four sample sequences and replacing all ambiguous bases against ‘N’. Gubbins (version 1.4.5, default parameters) [31] was used for recombination prediction based on the Mauve alignment. Predicted recombined regions (> 7,500 bp) were subsequently scanned with SeqSphere ^+^  against all cgMLST targets (using BLAST with thresholds 66% identity and 50% overlap) to reveal corresponding targets within the recombined region.

### Data availability

All raw reads generated were submitted to the European Nt Archive (http://www.ebi.ac.uk/ena/) of European molecular biology laboratory (EMBL) European Bioinformatics Institute (EBI) under the study accession number PRJEB12633. The cgMLST targets as well as the allelic profiles of each isolate were deposited at the cgMLST.org nomenclature server (http://www.cgmlst.org).

## Results

The outbreak occurred in the city of Warstein, Germany, in 2013 and involved 78 laboratory-confirmed LD cases [23]. Respiratory samples from 10 patients tested positive using the *L. pneumophila* and the Sg1-specific PCRs. From seven of these patients, eight clinical isolates were recovered. All these isolates were characterised as *L. pneumophila* Sg1, mAb-subgroup Knoxville, ST345 and regarded as being of the particular outbreak strain. This outbreak strain was also isolated from an industrial CT (source A; source designation as in [23], two WWTPs (source B and C) and the river Wester (source D) running through the town of Warstein.

In total, 42 strains were used in cgMLST analysis. These strains comprised the eight clinical isolates identified by conventional means as outbreak strain and 23 epidemiologically related environmental ST345 isolates. Furthermore, seven unrelated ST345 strains (six environmental and one clinical) as well as two reference genomes of the strains Corby and Alcoy 2300/99 were also included (Table 1). The seven unrelated ST345 isolates had all been deposited in the European SBT database [10] by the time the outbreak occurred. Furthermore, two environmental *L. pneumophila* Sg1 isolates of the same mAb-subgroup Knoxville as the outbreak strain but of a different but close genotype, ST600, were analysed. These ST600 isolates were found in higher numbers than the epidemic strain in all environmental samples taken during the outbreak but were not recovered from any of the clinical samples (Table 1) [25].

Although the original scheme as described by Moran-Gilad et al. [18] consists of 1,521 targets, some targets can happen to be absent in some strains. Therefore, a minimum spanning tree (MST) was constructed based on 1,475 targets of the cgMLST scheme that were present in all analysed genomes. Remarkably, the MST identified two clearly distinguishable clusters of the ST345 isolates obtained during the outbreak, hereafter referred to as ST345-A and ST345-B (Figure).

**Figure:**
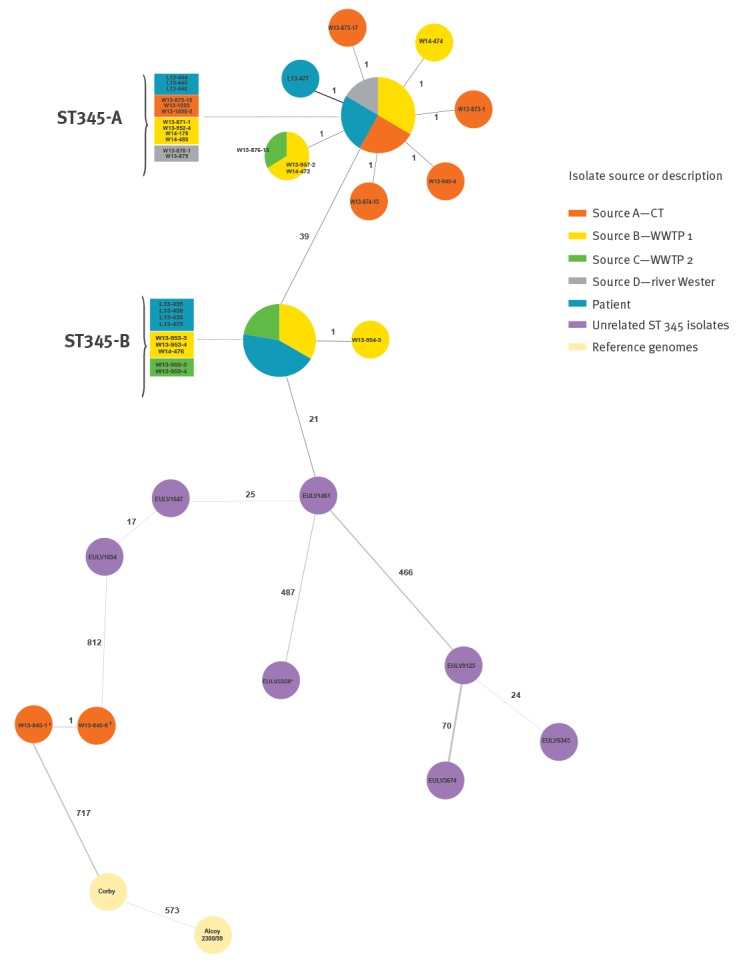
Analysis by minimum spanning tree based on 1,475 core genome multilocus sequence typing targets of isolates recovered in 2013 during a Legionnaires’ disease outbreak, Germany (n = 42 strains)

CT: cooling tower; WWTP: waste water treatment plant.

Numbers on lines represent the allelic differences between the isolates. All but four isolates in the Figure are sequence type (ST) 345. The two ST600 isolates are indicated with (§). All ST345 isolates which are unrelated to the outbreak and which serve as outgroup belong to the monoclonal antibody (mAb)-subgroup Knoxville, except EULV5358, which is mAb subgroup OLDA and which is indicated with (*).

Cluster ST345-A consisted of four clinical isolates (including two isolates from the same patient L13–445/-446) and 17 isolates recovered from all four putative environmental sources A–D. From these isolates, twelve showed no allelic difference and nine isolates differed each in a single allele from this central node. The remaining 10 ST345 outbreak isolates grouped as a separate cluster ST345-B, which differed in 39 alleles from ST345-A. Nine of the 10 ST345-B isolates showed an identical cgMLST profile and were isolated from four clinical and five environmental samples of sources B and C (both WWTPs). One environmental sample from source B showed one allele difference. Direct comparison of both clusters using all 1,521 cgMLST targets revealed in fact 40 alleles difference. The seven unrelated ST345 isolates were quite diverse revealing from 17 allelic differences between EULV1647 and EULV1654 up to 1,023 differences between EULV5358 and EULV3674. The unrelated ST345 isolate EULV1461 had only 21 allelic differences to the epidemic clone ST345-B. The two ST600 isolates showing only one allele difference from each other as well as the genomes of Corby and Alcoy 2300/99 clearly differ from the ST345 clones in more than 800 alleles (Figure).

We further investigated the differences between the two ST345 clusters in more detail. The aforementioned 40 different alleles are apparently located on two distinct neighbouring genomic regions including respectively 27 targets (recombination region 1; corresponding genes of reference strain Philadelphia-1 lpg2604–2636) and 13 targets (recombination region 2; lpg2666–2687) (Table 2).

**Table 2:** Core genome multilocus sequence typing targets differing between the two ST345 outbreak variants identified in a 2013 Legionnaires’ disease outbreak, Germany

Target^a^	Begin	End	Gene name	GenBank protein_ID	cgMLST allele number ST345-A	cgMLST allele number ST345-B	SNPs per target
Differing cgMLST targets in 42 kb recombination region (27 targets)							
lpg2604	2938631	2939434	NA^b^	YP_096609.1	1	4	11
lpg2606	2940021	2940887	NA^b^	YP_096611.1	1	4	5
lpg2607	2941026	2943062	*pepO*	YP_096612.1	1	4	21
lpg2608	2943206	2944120	*lpxC*	YP_096613.1	1	2	9
lpg2609	2944368	2945564	*ftsZ*	YP_096614.1	1	4	12
lpg2610	2945759	2947021	*ftsA*	YP_096615.1	1	4	26
lpg2612	2947732	2948838	*ddl*	YP_096617.1	1	4	16
lpg2614	2949745	2951154	*murC*	YP_096619.1	1	4	17
lpg2615	2951164	2952348	*ftsW*	YP_096620.1	3	5	14
lpg2616	2952345	2953688	*murD*	YP_096621.1	1	4	9
lpg2617	2953702	2954820	*mraY*	YP_096622.1	1	4	7
lpg2618	2954902	2956287	*murF*	YP_096623.1	1	4	13
lpg2619	2956480	2957259	NA^b^	YP_096624.1	1	4	7
lpg2620	2957264	2960758	NA^b^	YP_096625.1	1	5	63
lpg2621	2960933	2961613	NA^b^	YP_096626.1	1	4	9
lpg2622	2961715	2962776	NA^b^	YP_096627.1	1	4	13
lpg2623	2963086	2963898	NA^b^	YP_096628.1	1	4	12
lpg2624	2963973	2964455	*greA*	YP_096629.1	1	4	10
lpg2625	2964464	2967667	*carB*	YP_096630.1	1	4	77
lpg2626	2967794	2968066	NA^b^	YP_096631.1	1	4	22
lpg2627	2968179	2969360	NA^b^	YP_096632.1	1	4	85
lpg2629	2970147	2971217	NA^b^	YP_096634.1	1	4	13
lpg2630	2971214	2972215	NA^b^	YP_096635.1	1	4	15
lpg2631	2972463	2973947	*pepA*	YP_096636.1	1	4	25
lpg2633	2974501	2974818	NA^b^	YP_096638.1	3	5	5
lpg2635	2976210	2977781	*mviN*	YP_096640.1	1	4	26
lpg2636	2978139	2978405	*rpsT*	YP_096641.1	1	2	2
Differing cgMLST targets of 26 kb recombination region (13 targets)							
lpg2666	3013221	3014102	NA^b^	YP_096671.1	1	4	9
lpg2667	3014236	3015114	*rpoH*	YP_096672.1	1	4	5
lpg2668	3015387	3016316	*ftsX*	YP_096673.1	1	4	15
lpg2671	3018077	3019402	NA^b^	YP_096676.1	1	4	17
lpg2672	3019399	3020703	NA^b^	YP_096677.1	1	4	25
lpg2673	3020700	3021245	NA^b^	YP_096678.1	1	4	10
lpg2676	3022673	3023836	*dotB*	YP_096681.1	1	4	21
lpg2678	3025688	3026485	NA^b^	YP_096683.1	1	4	4
lpg2679	3026515	3027459	NA^b^	YP_096684.1	1	4	17
lpg2680	3027690	3028718	*murE3*	YP_096685.1	1	4	27
lpg2683	3030713	3032560	NA^b^	YP_096688.1	1	4	118
lpg2684	3032560	3033420	NA^b^	YP_096689.1	3	5	35
lpg2687	3037576	3038031	*icmV*	YP_096692.1	1	4	34

cgMLST: core genome multilocus sequence typing; ID: identity; SNP: single nt polymorphism; NA: not applicable.

^a^ Reference genome *Legionella pneumophila* strain Philadelphia-1; GenBank accession number NC_002942.5; cgMLST allele number for each target: ‘1’.

^b^ There is currently no assigned name for this gene.

The affected genes comprise virulence factors including genes of the Dot/Icm Type IV secretion system and genes involved in the muramyl synthesis [32,33]. In addition, a SNP analysis for these regions revealed several SNPs ranging from two to > 118 SNPs per gene with an average of two SNPs per 100 bp (Table 2). To investigate if the two regions indeed resulted from potential large recombination events, the genomes of four isolates were chosen, two from each cluster (ST345-A: W13–879–1/W13–952–4; ST345-B: L13–435/L13–473). The de novo assembled genomes were multiple aligned with Mauve, and searched for evidence of ‘import’ of divergent sequences from a distantly related source using Gubbins. Two large recombination regions of ca 42 and 26 kb size were predicted and subsequently screened for all cgMLST targets. The scanning procedure resulted exactly in the 27 and 13 cgMLST targets that were already detected as potentially recombinatory by SeqSphere ^+^. Finally, the 40 targets were compared against published genomes, thereby revealing that the ST345-A cluster differed from the ST36 strain Philadelphia-1 only in three of these (Table 2).

## Discussion

Here we present the results of the analysis of *L. pneumophila* ST345 strains isolated during the outbreak of Warstein 2013 [23] using a recently published cgMLST scheme. While less than 10% of reported LD cases occur in clusters and outbreaks [34], each outbreak must be regarded as a serious threat for public health since LD is a potentially life-threatening disease with case fatality rate of ca 10% [35]. Since the clinical picture of LD is not specific, the diagnosis always requires laboratory investigation. Of 78 epidemiologically and laboratory-confirmed cases of this large outbreak we were able to isolate the epidemic strain from seven patients. In three additional clinical samples that were culture-negative, nSBT allowed the complete ST in two (P13–308 and P13–733, Table 1), and a nearly complete allelic profile in the third (P13–402). Thus, we could detect the epidemic strain in samples of 10 patients (Table 1). The rate of complete or almost complete identified STs (10/78; 12%) is in the range as reported from other outbreaks [16,17,36]. However, there is a need to improve the recovery of clinical isolates in general in order to assign patients properly to an outbreak.

In the last 5 years, Legionnaires’ disease outbreak investigations have increasingly included WGS [15-17,19,20]. The main approach has been SNP-based, by mapping reads of clinical and environmental strains against a known reference genome. Although this enables precise differentiation between outbreak and non-outbreak isolates, the use of different reference genomes and mapping approaches makes SNP-based typing difficult to standardise. With the standardised generation, analysis and interpretation of WGS data and the establishment of a comprehensive bioinformatics pipeline and nomenclature, cgMLST allows to overcome this obstacle [18,19]. 

In this study, the application of cgMLST to a Legionnaires’ disease outbreak revealed two distinct clusters of the epidemic *L. pneumophila* clone, namely ST345-A and ST345-B, differing in 40 alleles. This difference clearly exceeds the preliminary threshold for a WGS cluster of four alleles difference, as shown previously [18,22]. Both clusters were indistinguishable by common gold standard methods and other typing methods [25]. Since strains in both clusters of the epidemic clone were almost equally distributed among clinical samples and epidemiologically linked to environmental strains by place and time of occurrence, we assume that this outbreak was caused by a single epidemic ST with two variants, which were already present in the environmental reservoirs before the outbreak occurred. 

Since the WGS analysis demonstrated a notable distance between the outbreak clusters, we closely examined the arrangement of the differing alleles. This analysis suggested that two major recombination events, most probably by horizontal gene transfer (HGT), may explain the differences between the two variants. Interestingly, the regions involved (42 and 26 kb) have already been reported as involved in a recombination event in a Spanish endemic clone of the same mAb-subgroup Knoxville [37]. The results of our investigation should serve as a note of caution for the use of WGS in outbreak investigations. Although gene-by-gene allele calling procedures like cgMLST inherently mitigate, in contrast to SNP calling procedures, against the effects of smaller recombination events, the method is prone to effects of large recombination events. Therefore, epidemiological information and/or compensation for recombination with methods as implemented in Gubbins or BratNextGen [38] are strongly recommended and, ideally, could be implemented in WGS-based typing and cgMLST standardised workflows.

An intriguing aspect would be the identification of the potential donor of the HGT regions. Comparison of the recombined regions with published genomes revealed a high similarity of the ST345-A variant to the *L. pneumophila* strain Philadelphia-1 (ST36), which was the causative agent of the first described outbreak in Philadelphia, 1976 [1,39]. Additional 19 isolates of the same ST36 described by Mercante et al. were identical to the Philadelphia-1 strain for these 40 targets (data not shown) [39]. Furthermore, three unrelated ST345 isolates (EULV1461; EULV1647 and EULV1654) shared the same 40 target alleles with the second cluster, ST345-B. We therefore assume that the ST345-B variant is the ancestral strain and the isolates of the ST345-A cluster evolved most probably by uptake of two large fragments from a donor strain in water systems that shared a high similarity with the Philadelphia strain genome. During the outbreak in Warstein environmental isolates of different sero- and mAb-subgroups were screened, but not tested in more depth and unfortunately not stored for later analysis which makes it impossible to identify the donor of the recombined regions.

Both ST345 clusters, ST345-A and ST345-B, which were identified during this outbreak, were identified in clinical and environmental sources. Several distinct potential environmental sources were confirmed that all harboured the epidemic clone including a CT, WWTPs and the river Wester that runs through the city of Warstein [23]. ST345-A isolates were detected in all four environmental reservoirs (sources A–D) while ST345-B isolates were found in two of them (sources B and C). However, all sources are located close to each other and are connected to or use the water of the river Wester (source D). The extensive epidemiological investigations indicated that this outbreak must be regarded as a multifactorial event with more than one sole source of contamination. It cannot be excluded that ST345-B inhabited the remaining two sources as well but might not have been sampled or isolated during the outbreak. The final clarification regarding which source might have contaminated other sources or whether one source is the main source of infection may never be solved in detail.

The suitability as well as the usability of a cgMLST to become a new standard typing method for *L. pneumophila* Sg1 isolates was recently discussed and requires further evaluation and refinement [18,40]. Core genome MLST combines a high discriminatory level with a standardised workflow and nomenclature which enables a global comparability of isolates. The latter is an important keynote for the surveillance and epidemiological investigation of LD wherein travel-associated infections play a significant role [7]. Thus, having the same typing tool is crucial. Combining knowledge on international level to define a robust scheme, a comprehensible workflow and uniform interpretation of data is mandatory. This is currently being mitigated by an international working group set up by the European Study Group for *Legionella* Infections (ESGLI) to ensure that cgMLST is globally implemented in a fit-for-purpose manner while maintaining backwards compatibility [41].

## Conclusion

Application of the cgMLST scheme for *L. pneumophila* demonstrated its usability during outbreak investigations. Core genome MLST showed a superior discriminatory power when compared with current gold standard typing methods, allowing for a higher resolution which resulted in finding that the epidemic strain split up into two variants. Furthermore, cgMLST indicated horizontal gene transfer as potential reason for the difference between both variants. This was confirmed by additional bioinformatics analyses. The value of classical epidemiological data was reinforced during the outbreak investigation, as such data anchored the isolates in time and space. These epidemiological data supported the findings that the large outbreak of LD in Warstein was caused by two variants, ST345-A and ST345-B, of the same ST345 clone. In the WGS era, cgMLST allows for a standardised workflow and nomenclature with high resolution and can even identify recombination events when allelic differences are clustered. However, the establishment of a globally uniform scheme needs to be well communicated and orchestrated in order to be cost-efficient and fit-for-purpose.
